# COVID-19’s natural course among ambulatory monitored outpatients

**DOI:** 10.1038/s41598-021-89545-1

**Published:** 2021-05-12

**Authors:** Barbora Weinbergerova, Jiri Mayer, Stepan Hrabovsky, Zuzana Novakova, Zdenek Pospisil, Lucie Martykanova, Katerina Hortova, Lucie Mandelova, Karel Hejduk, Renata Chloupková, Michal Pospisil, Martina Doubkova, Vladimir Marek, Renata Novotna, Martin Dolecek, Hana Matejovska Kubesova, Kristian Brat, Radana Parizkova, Petr Husa, Marek Mechl, Zdenek Kral, Martina Lengerova

**Affiliations:** 1 Department of Internal Medicine, Hematology and Oncology, Masaryk University and University Hospital Brno, Brno, Czech Republic; 2 Department of Mathematics and Statistics, Faculty of Science, Masaryk University, Brno, Czech Republic; 3 Institute of Health Information and Statistics of the Czech Republic, Prague, Czech Republic; 4 Institute of Biostatistics and Analyses, Faculty of Medicine, Masaryk University, Brno, Czech Republic; 5 Department of Nursing and Midwifery, Faculty of Medicine, Masaryk University, Brno, Czech Republic; 6 Department of Respiratory Diseases, Masaryk University and University Hospital Brno, Brno, Czech Republic; 7 Department of Internal Medicine, Geriatrics and Practical Medicine, Masaryk University and University Hospital Brno, Brno, Czech Republic; 8 General Practitioner’s Office, Berkova 2390/107, 61200 Brno, Czech Republic; 9 Department of Anesthesiology and Intensive Care Medicine, Masaryk University and University Hospital Brno, Brno, Czech Republic; 10 Department of Infectious Diseases, Masaryk University and University Hospital Brno, Brno, Czech Republic; 11 Department of Radiology and Nuclear Medicine, Masaryk University and University Hospital Brno, Brno, Czech Republic

**Keywords:** Viral infection, Genetic testing, Comorbidities, Respiratory distress syndrome, Risk factors, Lifestyle modification, Preventive medicine, Respiratory signs and symptoms, Patient education, Epidemiology, Population screening

## Abstract

Research objective was to detail COVID-19’s natural trajectory in relation to the Czech population’s viral load. Our prospective detailed daily questionnaire-based telemonitoring study evaluated COVID-19’s impact among 105 outpatients. In accordance with government quarantine requirements, outpatients were divided into a cohort with two negative tests at the end of the disease (40 patients) and a cohort with a new algorithm (65 patients) following a 14-day quarantine. Median follow-up differed significantly between the 2 groups (23 days vs. 16 days). Only 6% of patients were asymptomatic during the entire telemonitoring period. Another 13% of patients were diagnosed asymptomatic, as suspected contacts, yet later developed symptoms, while the remaining 81% were diagnosed as symptomatic on average 6 days following symptom onset. Telemonitoring enabled precise symptom status chronicling. The most frequently reported complaints were fevers, respiratory issues, and anosmia. Six patients were eventually hospitalized for complications detected early after routine telemonitoring. During the extended follow-up (median 181 days), anosmia persisted in 26% of patients. 79% of patients in the new quarantine algorithm cohort reported no symptoms on day 11 compared to just 56% of patients in the two negative test cohort upon first testing negative (median–19 days). The highest viral load occurred within 0–2 days of initial symptom onset. Both the PCR viral load and two consecutive PCR negative sample realizations indicated high interindividual variability with a surprisingly fluctuating pattern among 43% of patients. No definitive COVID-19 symptoms or set of symptoms excepting anosmia (59%) and/or ageusia (47%) were identified. No preexisting medical conditions specifically foreshadowed disease trajectory in a given patient. Without a PCR negativity requirement for quarantine cessation, patients could exhibit fewer symptoms. Our study therefore highlights the urgent need for routine ambulatory patient telemedicine monitoring, early complication detection, intensive mass education connecting disease demeanor with subsequent swift diagnostics, and, notably, the need to reevaluate and modify quarantine regulations for better control of SARS-CoV-2 proliferation.

## Introduction

Coronavirus Disease 2019 (COVID-19), triggered by coronavirus SARS-CoV-2, is a novel disease that spread from China to virtually the entire world in early 2020^1,2^. This unprecedented disease has seriously impacted global health, social dictums, and worldwide economics, resulting in a great number of articles being published describing pathogenesis, diagnostics, clinical course, treatment, and vaccine development^2–12^. Despite the degree of knowledge, many concerns remain, especially in the area targeting the natural course of the disease. An exact description of individual symptoms and their duration is still lacking^3–7,12–15^. Concurrently, working with imprecise data has led to article retractions, even from prestigious journals^16^.

Initial clinical trials from China and Italy recorded an alarmingly high rate of severe pneumonia caused by SARS-CoV-2^17,18^. Additional studies describe pulmonary infiltrates in asymptomatic patients^5,19–22^. It seems probable that the disease may have a dissimilar course among divergent races^23^. Autopsy investigations indicate evidence of the virus affecting a wide range of different organs, causing a number of serious complications^24^. Certain risk factors increasing the likelihood of a serious disease course have recently been identified^25^.

Our prospective cohort study was developed during the first wave of the coronavirus pandemic in the Czech Republic when there was a demand to delineate the clinical picture of a disease decimating the Czech population. Czech outpatient care had generally been significantly reduced, and no systematic care procedure was established for COVID-19 positive outpatients not requiring hospitalization. Patients had been advised to call an emergency service if and when their health was deteriorating.

In an emergency situation, sophisticated telemedicine is of great importance for healthcare^26^. During the coronavirus pandemic, its significance became clearly evident. Moreover, for successful pandemic management, goals must include effective education and citizen cooperation coordinated by supportive state governing bodies.

Our objectives were as follows:

1. To map in detail the natural course of the disease in selected Czech patients who were primarily ambulatory monitored in-home care, not requiring admission to the hospital at the time of diagnosis. Telemedicine and protocol-based management with the guidance of professional healthcare specialists were employed.
2. To detect early imminent disease complications with a specific focus on pneumonia, which could be underestimated by patients without regular telemonitoring and implementation of rapid diagnostics and treatment.
3. To describe viral load kinetics in relation to the spectrum of clinical symptoms.
4. To develop a simple model algorithm for telemedicine monitoring, which could become a national standard for general practitioners.

## Methods

### Study population

Our prospective observational standardized study "COVID-JMK-20" recruitment period was ongoing from April 20, 2020 to September 2, 2020. Participation was offered to all adults who encountered our mobile testing location at the University Hospital. Study project was approved by the Ethics Committee of the University Hospital Brno (Number 01-130520/EK). All research was undertaken in accordance with relevant guidelines and regulations. All patients signed an informed consent form.

### Monitoring schedule

All outpatients were monitored daily, immediately following their first SARS-CoV-2 positivity, via a predefined questionnaire (see Supplementary Appendix 1 in Supplementary Information). Later, if conditions stabilized, monitoring intervals could be prolonged. In total, incidence and duration of 18 disease symptoms were recorded. Importantly, we also included symptoms present prior to patient’s diagnostic polymerase chain reaction (PCR) test. Phone interviews were conducted by two workers with medical education backgrounds. If health conditions deteriorated during our follow-up (e.g. respiratory distress with suspicion of COVID-19 pneumonia), patients would be contacted by one of the University Hospital Brno study team’s three physicians, who evaluated severity status and potential need for further examination and/or hospitalization.

Although our comprehensive project was designed for one year of inclusive patient clinical and immunological follow-up, primary analysis focused on acute symptom initial phase evaluations. Nonetheless, patient telemonitoring continued until quarantine was completed in accordance with Ministry of Health regulations. During the initial period from 20 April 2020 to 7 July 2020, a 14-day quarantine was required after the first positive PCR test and consequently terminated after patients exhibited no symptoms for at least 3 consecutive days and twice tested negative with PCR tests performed minimally 24 h apart^27^. During study enrollment, government regulations were revised on July 8, 2020, when patients, following a minimum 14-day quarantine including at least 4 asymptomatic days prior to quarantine termination would be considered non-infectious without the need for PCR test negativity^28^.

**Figure 1:**
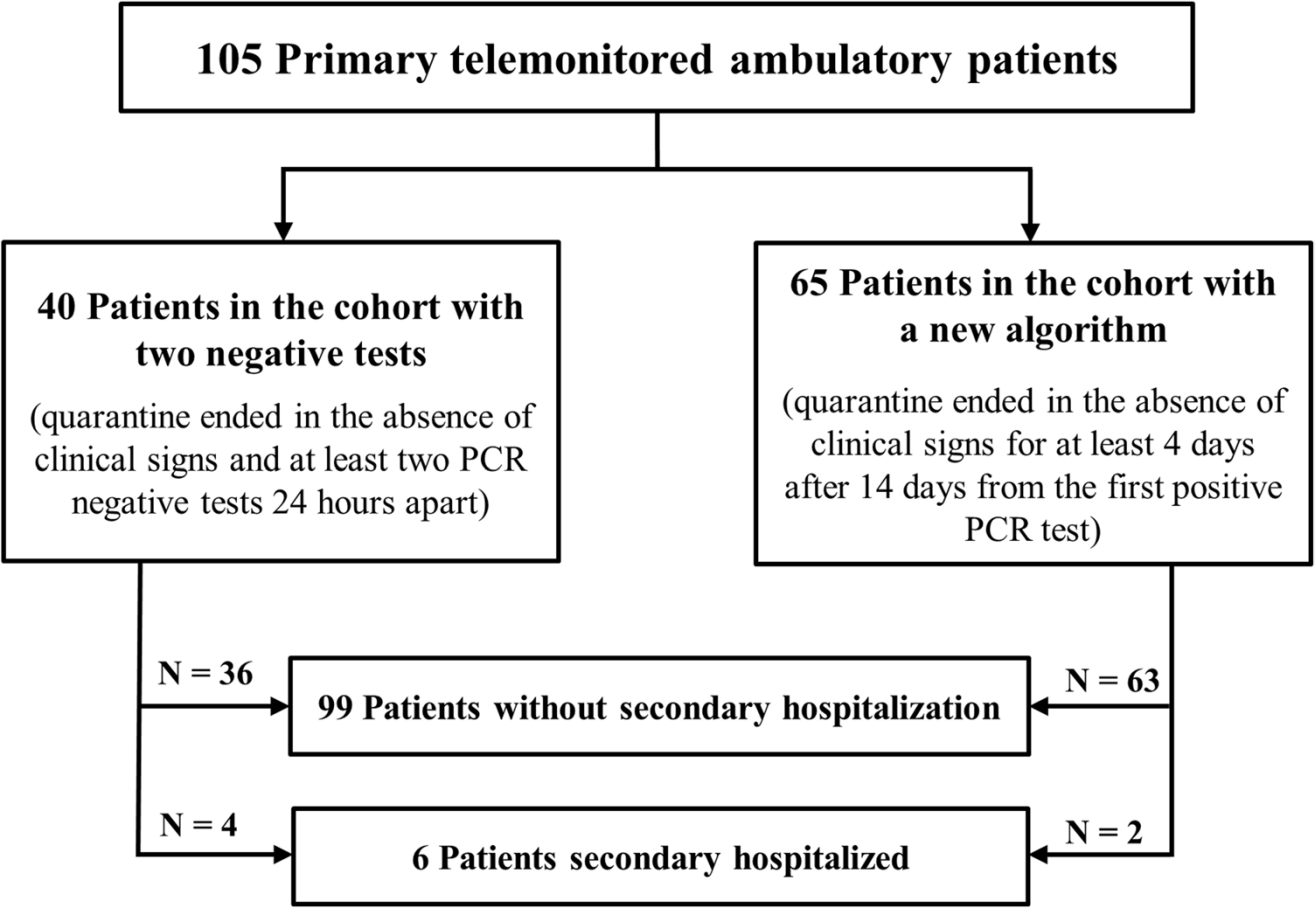
Diagram of patient selection algorithm for the analysis, which aims to describe symptoms in ambulatory monitored outpatients. *PCR* polymerase chain reaction.

A descriptive analysis was initially performed on all outpatients (n = 105) and then separately for 2 divided cohorts: "Cohort with Two Negative Tests" enrolled through 7 July 2020 vs. "Cohort with New Algorithm" participating from 8 July 2020. During our initial study phase, symptom length was evaluated only among the first cohort with two negative tests, whereas symptom duration assessment in the new algorithm cohort was deemed immaterial owing to limited monitoring capacity. Subsequently, we recorded symptom duration throughout extended monitoring. Regarding time classification of patient symptoms, day 0 was determined either as the day of initial symptom onset or the time of first positive sample, whichever came first. Our patient selection algorithm is summarized in Fig. 1.

### SARS-CoV-2 detection

Viral RNA was extracted from 300 µl of a nasopharyngeal swab sample in a viral transport medium using the LabTurbo Viral DNA/RNA Mini Kit (Taigen Bioscience, Taiwan). Reverse transcription PCR was conducted with gb SARS-CoV-2 Multiplex (Generi Biotech, Czech Republic) according to manufacturers’ instructions. Result was considered valid only when cycle threshold (Ct) value of the reference gene was ≤ 38.0. Result was considered positive when both target genes (E and RdRP) were detected. If only one of the target genes was positive, the sample was reanalyzed. Samples with a Ct value ≥ 50.0 were interpreted as negative. The viral elimination course was evaluated only in the cohort with two negative tests, and for this particular analysis, each patient’s day 0 was determined as the day of the first positive sample.

### Statistical analysis

Basic statistical methods describing absolute and relative frequency for categorical variables, mean and median, supplemented by minimum and maximum for continuous variables, respectively, were used. Categorical parameters relation was evaluated using Pearson’s Chi-squared and Fisher’s exact tests. Continuous variables were compared using Mann–Whitney *U* test and Kruskal–Wallis rank sum test. For all analyses, α = 0.05 was used as a level of statistical significance, unless otherwise stated. For statistical analysis, software R version 3.5.2 was used. Non-linear dependencies of variables were visualized by the nonparametric regression curves obtained using the Gaussian kernel. For the circular visualization of symptoms’ co-occurrence, the package “Circlize version 0.4.11” was used.

## Results

In the Czech Republic, a total of 558,650 unique patients were tested via nasopharyngeal swab during our study, and 19,004 (3%) were deemed COVID-19 positive, engendering a 5.9% hospitalization rate and 1.3% fatality rate^29^. Initially, our study recruitment was very successful with significant participation. Enrollment dropped dramatically, however, as the first wave of infections subsided in the Czech Republic, and fewer medical students were available at mobile sampling points to explain study principles and advantages. Study involvement later accelerated in the second half of August 2020, when numbers of infected people notably increased. At the University Hospital, during the study period, SARS-CoV-2 positivity was detected in 5% (n = 594) of 11,469 examined patients, and 87% (n = 517) were COVID-19 positive outpatients. In total, 105 outpatients (20% of University Hospital’s COVID-19 positive outpatients) agreed to participate in our study. A total of 1223 phone interviews were conducted (mean 12; median 11; min 1, max 25).

### Demography and comorbidities

Among 105 cohort outpatients, the mean age was 40 years with slightly more women (52%). Eighty-four (80%) patients had some comorbidity with the following frequency breakdown: n = 1, 37%; n = 2, 24%; n = 3, 8%; n = 4, 5%; n = 5, 5%; n = 6, 1%, with the most frequent being allergy (43%) and hypertension (24%), (Tables 1, 2). No significant difference was recorded between the two negative test cohort (40 patients; 38%) and the new algorithm cohort (65 patients; 62%) with regard to baseline characteristics and comorbidities (Table 1).

**Table 1:** Characteristics of outpatients enrolled in the study.

N (%)		Total	Cohort		p-value
			Two negative tests	New algorithm	
Number of patients		105 (100)	40 (38.1)	65 (61.9)	NA
Gender	Male	50 (47.6)	20 (50.0)	30 (46.2)	0.841
	Female	55 (52.4)	20 (50.0)	35 (53.8)	
Age	Mean; median (min–max)	40; 37 (18–78)	39; 39 (18–64)	41; 37 (18–78)	0.692
	< 30	30 (28.6)	14 (35.0)	16 (24.6)	0.078
	30–40	28 (26.7)	7 (17.5)	21 (32.3)	
	40–50	18 (17.1)	8 (20.0)	10 (15.4)	
	50–60	16 (15.2)	9 (22.5)	7 (10.8)	
	≥ 60	13 (12.4)	2 (5.0)	11 (16.9)	
Weight (kg)	Mean; median (min–max)	79; 77 (42–140)	78; 80 (46–130)	79; 76 (42–140)	0.908
Height (cm)	Mean; median (min–max)	173; 175 (150–199)	172; 174 (156–192)	174; 175 (150–199)	0.385
Body mass index	Mean; median (min–max)	26.1; 24.9 (17.5–52.1)	26.3; 24.8 (18.4–38.9)	25.9; 25.0 (17.5–52.1)	0.644
Comorbidities	Diabetes mellitus	7 (6.7)	2 (5.0)	5 (7.7)	0.706
	Hypertension	25 (23.8)	9 (22.5)	16 (24.6)	0.999
	Smoking	13 (12.4)	8 (20.0)	5 (7.7)	0.074
	Oncological disease	11 (10.5)	6 (15.0)	5 (7.7)	0.326
	Autoimmune disease	6 (5.7)	4 (10.0)	2 (3.1)	0.198
	Allergy	45 (42.9)	14 (35.0)	31 (47.7)	0.228
	Other	45 (42.9)	16 (40.0)	29 (44.6)	0.688
Time from the first positive symptom to the first positive sampling (days)^a^	Mean; median (min; max)	6.1; 4.0 (0.0; 36.0)	6.8; 4.0 (0.0; 36.0)	5.7; 4.0 (0.0; 32.0)	1.0
Thermometer type used	Mercury	27 (42.9)	13 (52.0)	14 (36.8)	0.254
	Digital	23 (36.5)	7 (28.0)	16 (42.1)	
	Not specified	13 (20.6)	5 (20.0)	8 (21.1)	–
Length of telemonitoring	Mean; median (min; max)	18.4; 14.0 (0.0; 54.0)	22.7; 19.5 (7.0; 54.0)	15.7; 12.0 (0.0; 44.0)	0.001

^a^Only patients with any symptom at the time of sampling included (i.e. without 14 asymptomatic patients at the time of sampling).

**Table 2:** Fifty-nine other comorbidities in 45 outpatients.

Other comorbidities^a^	N	%
Hypercholesterolemia	6	5.7
Arrhythmias, tachycardia, carditis	5	4.8
Mental illness	5	4.8
Thyroid hypofunction	4	3.8
Asthma bronchiale	3	2.9
Gastroesophageal reflux	2	1.9
Acute gouty arthritis	2	1.9
Unspecified venous disorders	2	1.9
Leg ulcers	1	1.0
Spinal dysraphism	1	1.0
Epilepsy	1	1.0
Chronic pancreatitis	1	1.0
Anemia	1	1.0
Intermittent hepatopathy	1	1.0
Oesophageal hernia	1	1.0
Mononucleosis	1	1.0
Colostomy	1	1.0
Nephrostomy	1	1.0
Migraine	1	1.0
Obesity	1	1.0
Molds (feet, hands)	1	1.0
Chronic rhinitis	1	1.0
Polycystic ovaries	1	1.0
Coagulopathy	1	1.0
Raynaud’s phenomenon	1	1.0
Neuropathy	1	1.0
Duodenal ulcer	1	1.0
Hemicolectomy	1	1.0
Hepatitis B	1	1.0
Gluten intolerance	1	1.0
Prostate disease	1	1.0
Leukopenia	1	1.0
Pulmonary embolism	1	1.0
Artificial heart valve	1	1.0
Other	4	3.8

^a^One patient may have had multiple comorbidities.

### Secondary hospitalized outpatients

A telephone interview conducted by a doctor was necessary for 10 (10%) outpatients, from which 7 (7%) required examination and six (6%) were eventually hospitalized with a 7 day median following diagnostic test and a 4 day median hospital stay (Table 3). The male majority (83%) of hospitalized patients were admitted to the hospital with a median age of 56 years and a median of 2 comorbidities. Symptom frequency and median duration were as follows: Fever (83%; 9 days), dyspnea (67%; 2 days), cough (67%; 6 days), and diarrhea (33%; 1 day). In relation to COVID-19, pneumonia mandated hospitalization in 2 patients, diarrhea in 2 patients, atypical thoracalgia in 1 patient, and dyspnea with fever in 1 patient. We evaluated the disease course as mild in 4 patients and moderate in 2 patients. One patient was treated with remdesivir and one patient with a combination of hydroxychloroquine and azithromycin. No patient died. Table 3 provides a detailed description of secondary hospitalized outpatients.

**Table 3:** Baseline characteristics of 6 outpatients with SARS-CoV-2 positivity during secondary hospitalization.

ID no	Sex	Age (years)	Days to admission from 1st positivity	Length of hospitalization (days)	Comorbidity	Reason of hospitalization	No of febrile days	Pneumonia	COVID-19 severity	Comment
2	M	65	6	4	Metabolic syndrome	Thoracalgia	13	No	Mild	Therapy: HCQ + AZM
16	F	27	4	4	Bronchial asthma	Fever, diarrhea	1	No	Mild	–
23	M	34	5	2	GERD	Dyspnea, epigastric pain, diarrhea	NA	No	Mild	–
57	M	52	14	13	Metabolic syndrome, CRC, sigmoidostomy, bilateral nephrostomy for nephrolithiasis	Dyspnea, fever, nephrostomy obstruction	1	No	Mild	Therapy: ATB
65	M	60	7	4	AHT, HLP	Fever, dyspnea, cough	9	Yes	Moderate	
120	M	66	10	9	AHT, PE, hyperuricemia	Fever, dyspnea, cough	10	Yes	Moderate	Therapy: RDV + DEX + ATB

*SARS-CoV-2* severe acute respiratory syndrome coronavirus 2, *ID no* identification number, *COVID-19* coronavirus disease-19, *GERD* gastroesophageal reflux disease, *AHT* arterial hypertension, *NA* not applicable, *DEX* dexamethasone, *ATB* antibiotics, *RDV* remdesivir, *HCQ* hydroxychloroquine, *AZM* azithromycin, *CRC* colorectal carcinoma, *HLP* hyperlipidemia, *PE* pulmonary embolism.

For suspected pneumonia, a 50-year-old obese woman with dyspnea and cough from the two negative test cohort underwent chest computer tomography. There was no pulmonary pathological finding apart from solitary cervical lymphadenopathy. After symptoms persisted for 38 days and PCR test result was negative, further diagnostic procedures were performed. Eventually, Castleman disease turned out to be the reason for persistent clinical symptoms.

### COVID-19 outpatient symptomatology

Among 99 (94%) of symptomatic outpatients, symptom median number was 7 (mean 7.0; min 0, max 17). During diagnostic test sampling, 14 (13%) patients were pre-symptomatic and developed some symptoms during disease progression. Only 6 (6%) of outpatients were completely asymptomatic throughout the episode. All evaluated symptoms are shown in Table 4. Time distribution of symptoms is detailed in Table 5.

**Table 4:** Incidence and duration of COVID-19 symptoms in outpatients.

Type of symptom	All outpatients	Cohort with two negative tests		Cohort with new algorithm	p-value
	N (%)	N (%)	Duration (days) mean; median (min–max)	N (%)	
Any clinical symptom	99 (94.3)	37 (92.5)	NA	56 (95.4)	0.672
Fever ≥ 37 °C	63 (60.0)	25 (62.5)	8.0; 5.0 (1.0–42.0)	38 (58.5)	0.838
Dry cough	45 (42.9)	19 (47.5)	16.4; 12.0 (1.0–55.0)^a^	26 (40.0)	0.543
Wet cough	42 (40.0)	15 (37.5)	13.5; 14.0 (2.0–26.0)^a^	27 (41.5)	0.838
Respiratory tract infection signs	74 (70.5)	27 (67.5)	15.8; 10.0 (2.0–56.0)^a^	47 (72.3)	0.662
Ageusia	49 (46.7)	19 (47.5)	23.4; 26.0 (2.0–55.0)^a^	30 (46.2)	1.0
Anosmia	62 (59.0)	23 (57.5)	24.2; 26.0 (2.0–55.0)^a^	39 (60.0)	0.840
Headache	61 (58.1)	22 (55.0)	8.1; 5.0 (1.0–31.0)^a^	39 (60.0)	0.686
Musculoskeletal pain	58 (55.2)	18 (45.0)	9.7; 5.0 (1.0–54.0)	40 (61.5)	0.110
Diarrhea	21 (20.0)	10 (25.0)	4.2; 2.0 (1.0–22.0)	11 (16.9)	0.327
Abdominal pain	12 (11.4)	7 (17.5)	2.9; 2.0 (1.0–7.0)	5 (7.7)	0.205
Anorexia	40 (38.1)	14 (35.0)	7.8; 5.0 (1.0–37.0)	26 (40.0)	0.682
Vomiting	1 (1.0)	0 (0.0)	NA	1 (1.5)	NA
Breath difficulties	14 (13.3)	8 (20.0)	13.9; 7.0 (2.0–55.0)^a^	6 (9.2)	0.143
Dyspnea	10 (9.5)	4 (10.0)	23.0; 16.0 (5.0–55.0)^a^	6 (9.2)	1.0
Shortness of breath	3 (2.9)	1 (2.5)	17.0; 17.0 (17.0–17.0)^a^	2 (3.1)	1.0
Tachypnea	3 (2.9)	2 (5.0)	3.5; 3.5 (2.0–5.0)	1 (1.5)	0.556
Thoracalgia	13 (12.4)	8 (20.0)	12.3; 5.0 (2.0–52.0)^a^	5 (7.7)	0.074
Dry skin	1 (1.0)	1 (2.5)	19; 19 (19.0–19.0)^a^	0 (0)	0.381
Other symptoms	76 (72.4)	24 (60.0)	NA	52 (80.0)	**0.042**

*COVID-19* coronavirus disease-19, *NA* not applicable.

^a^Symptom continued on the last phone call at least in one patient. Telemonitoring was ended due to double PCR negative testing.

**Table 5:** Symptoms’ onset during COVID-19.

Type of symptom	All outpatients		Cohort with two negative tests	
	Start (days) Mean; median (min–max)	Stop (days) Mean; median (min–max)	Start (days) Mean; median (min–max)	Stop (days) Mean; median (min–max)
Fever ≥ 37 °C	2.4; 0.0 (0.0–30.0)	9.4; 5.0 (1.0–42.0)	1.7; 0.0 (0.0–23.0)	9.7; 6.0 (1.0–42.0)
Dry cough	3.2; 1.0 (0.0–20.0)	16.5; 13.0 (2.0–56.0)*	1.5; 0.0 (0.0–11.0)	18.0; 14.0 (2.0–56.0)*
Wet cough	5.6; 2.0 (0.0–35.0)	15.9; 16.0 (3.0–44.0)*	6.1; 1.0 (0.0–35.0)	19.6; 20.0 (6.0–37.0)*
Respiratory tract infection signs	3.0; 1.0 (0.0–26.0)	14.5; 11.0 (3.0–56.0)*	3.4; 1.0 (0.0–26.0)	19.3; 16.0 (4.0–56.0)*
Ageusia	4.6; 4.0 (0.0–27.0)	19.2; 15.0 (4.0–56.0)*	3.0; 2.0 (0.0–14.0)	26.4; 26.0 (6.0–56.0)*
Anosmia	4.5; 4.0 (0.0–27.0)	20.; 17.0 (3.0–56.0)*	3.1; 4.0 (0.0–14.0)	27.3; 26.0 (8.0–56.0)*
Headache	3.3; 0.0 (0.0–48.0)	10.7; 9.0 (2.0–56.0)*	5.8; 1.0 (0.0–48.0)	13.9; 11.0 (2.0–56.0)*
Musculoskeletal pain	2.1; 0.0 (0.0–22.0)	10.2; 7.0 (2.0–55.0)	2.2; 0.0 (0.0–10.0)	11.8; 9.0 (2.0–55.0)
Diarrhea	4.7; 2.0 (0.0–24.0)	9.3; 5.0 (2.0–32.0)	6.5; 2.5 (0.0–24.0)	10.7; 5.0 (2.0–32.0)
Abdominal pain	9.0; 9.5 (0.0–24.0)	14.7; 14.0 (4.0–33.0)	8.7; 9.0 (1.0–24.0)	11.6; 11.0 (4.0–27.0)
Anorexia	4.4; 1.0 (0.0–30.0)	11.9; 10.0 (3.0–38.0)	3.9; 0.5 (0.0–24.0)	11.7; 9.0 (3.0–38.0)
Vomiting	1.0; 1.0 (1.0–1.0)	3.0; 3.0 (3.0–3.0)	NA	NA
Breath difficulties	5.1; 1.5 (0.0–22.0)	16.1; 13.5 (5.0–56.0)*	3.4; 1.0 (0.0–14.0)	17.3; 13.5 (6.0–56.0)*
Dyspnea	8.3; 5.5 (0.0–29.0)	20.8; 15.0 (7.0–56.0)*	0.75; 0.5 (0.0–2.0)	23.8; 16.0 (7.0–56.0)*
Shortness of breath	10.7; 10.0 (0.0–22.0)	19.0; 17.0 (15.0–25.0)*	0.0; 0.0 (0.0–0.0)	17.0; 17.0 (17.0–17.0)*
Tachypnea	8.7; 2.0 (2.0–22.0)	12.0; 7.0 (4.0–25.0)	2.0; 2.0 (2.0–2.0)	5.5; 5.5 (4.0–7.0)
Thoracalgia	7.2; 6.0 (0.0–28.0)	16.1; 11.0 (6.0–53.0)*	6.3; 1.5 (0.0–28.0)	18.5; 12.5 (6.0–53.0)*
Dry skin	37; 37 (37.0–37.0)	56; 56 (56.0–56.0)*	37; 37 (37.0–37.0)	56; 56 (56.0–56.0)*

For each patient, day 0 was determined either as the day of first symptom onset, or the day of the first positive sample, whichever came first, although the majority of patients generally had symptoms before the first positive test (see Fig. 6). Six completely asymptomatic patients are not included in this Table.

*COVID-19* coronavirus disease-19, *NA* not applicable.

^a^Symptom continued on the last phone call at least in one patient. Telemonitoring was ended due to double PCR negative testing.

#### COVID-19 symptom frequency

Regarding symptom incidence, most common reported symptoms were: General symptoms of respiratory tract infection (RTI) (71%), fatigue (65%), fever (60%) with a median of 37.6 °C, anosmia (59%), headache (58%), musculoskeletal pain (55%), ageusia (47%), and dry cough (43%) (Table 4). Females reported a higher frequency of anosmia (66% vs. 52%; p = 0.172) as well as younger patients (median age of patients with anosmia vs. without anosmia was 34 vs. 47 years; p = 0.016). Other less frequent symptoms were noted in Table 6.

**Table 6:** Incidence of other COVID-19 symptoms in 76 outpatients.

Symptom	N^a^	% from all outpatients
Fatigue	68	64.8
Eye pain	14	13.3
Nausea	12	11.4
Excessive sweating	11	10.5
Dizziness	8	7.6
Chills	8	7.6
Stuffy nose	6	5.7
Bad taste in the mouth	4	3.8
Burning nose	4	3.8
Conjunctivitis	4	3.8
Sneezing	3	2.9
Dry mouth	3	2.9
Burning eyes	2	1.9
Pruritus	2	1.9
Stomach pain	1	1.0
Swollen nodes	1	1.0
Herpes	1	1.0
Sparse stools	1	1.0
Head pressure	1	1.0
Tremor	1	1.0

*COVID-19* coronavirus disease-19.

^a^One patient may have had multiple symptoms.

#### Length of COVID-19 symptoms

**Figure 2:**
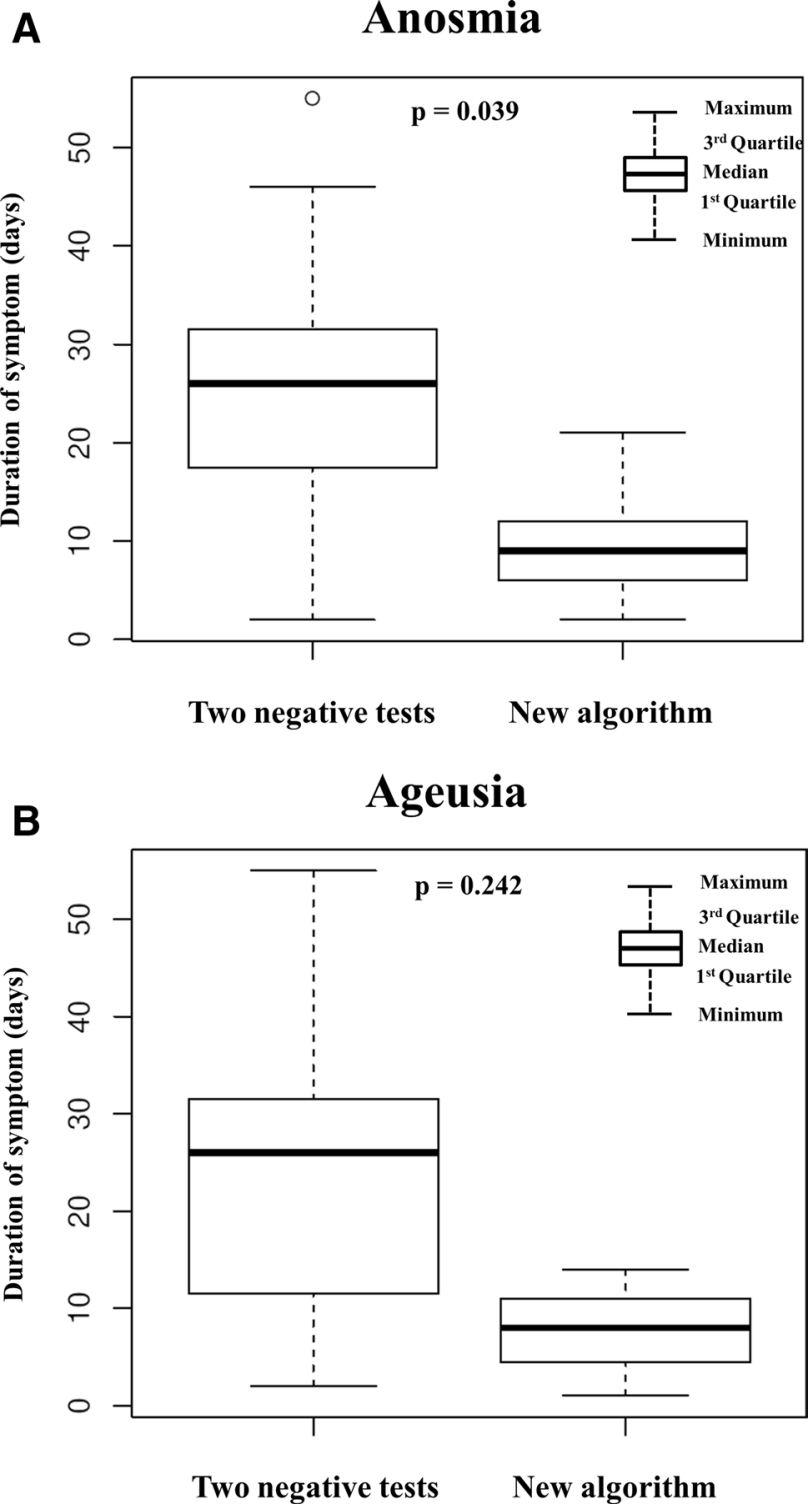
(**A**) Anosmia duration in the cohort with two negative tests and the cohort with new algorithm. The box plots show anosmia duration in 23 patients in the cohort with two negative tests vs. 36 patients in the cohort with new algorithm. Three patients with a more severe disease course and longer follow-up for other objective complications were excluded from the new algorithm cohort. The small circle at the top of the graph marks an outlier. (**B**) Ageusia duration in the cohort with two negative tests and the cohort with new algorithm. The box plots show ageusia duration among 19 patients in the cohort with two negative tests vs. 27 patients in the cohort with new algorithm. Three patients with a more severe disease course and longer follow-up for objective complications were excluded from the new algorithm cohort.

Evaluated only in the two negative tests cohort, the shortest duration of symptoms with a median of up to 5 days included fever, headache, musculoskeletal pain, diarrhea, anorexia, tachypnea, thoracalgia, and abdominal pain. Conversely, breathing difficulties, general RTI symptoms, dry and wet cough, dyspnea, shortness of breath, anosmia, and ageusia had a longer median duration exceeding 10 days (Table 4). During telemonitoring termination owing to double PCR negativity, certain symptoms still persisted in 41% of patients. Regarding comparison of the length of anosmia and ageusia between the two outpatient cohorts, we observed a longer median duration of both symptoms in the cohort with two negative tests (anosmia 26 days vs. 9 days; p = 0.039; ageusia 26 days vs. 8 days; p = 0.242, respectively) (Fig. 2).

#### Time distribution of COVID-19 symptoms

In terms of symptom time distribution related to disease onset, fever, headache and musculoskeletal pain, practically appeared at median day zero from disease onset. Subsequent symptoms comprising dry and wet cough, general RTI symptoms, diarrhea, anorexia, breathing difficulties, and tachypnea were later reported with a median of 1–2 days following disease onset. Finally, late symptoms with a median onset of more than 2 days involved anosmia, ageusia, abdominal pain, dyspnea, and shortness of breath (Table 5). Symptom termination time also varied. Certain symptoms disappeared median 10 days after disease onset (fever, headache, musculoskeletal pain, diarrhea, anorexia, tachypnea), while most symptoms lasted more than median 11 days following disease onset (anosmia, ageusia, dry and wet cough, general RTI symptoms, abdominal pain, dyspnea, breathing difficulties, and shortness of breath).

#### Co-occurrence of clinical symptoms

**Figure 3:**
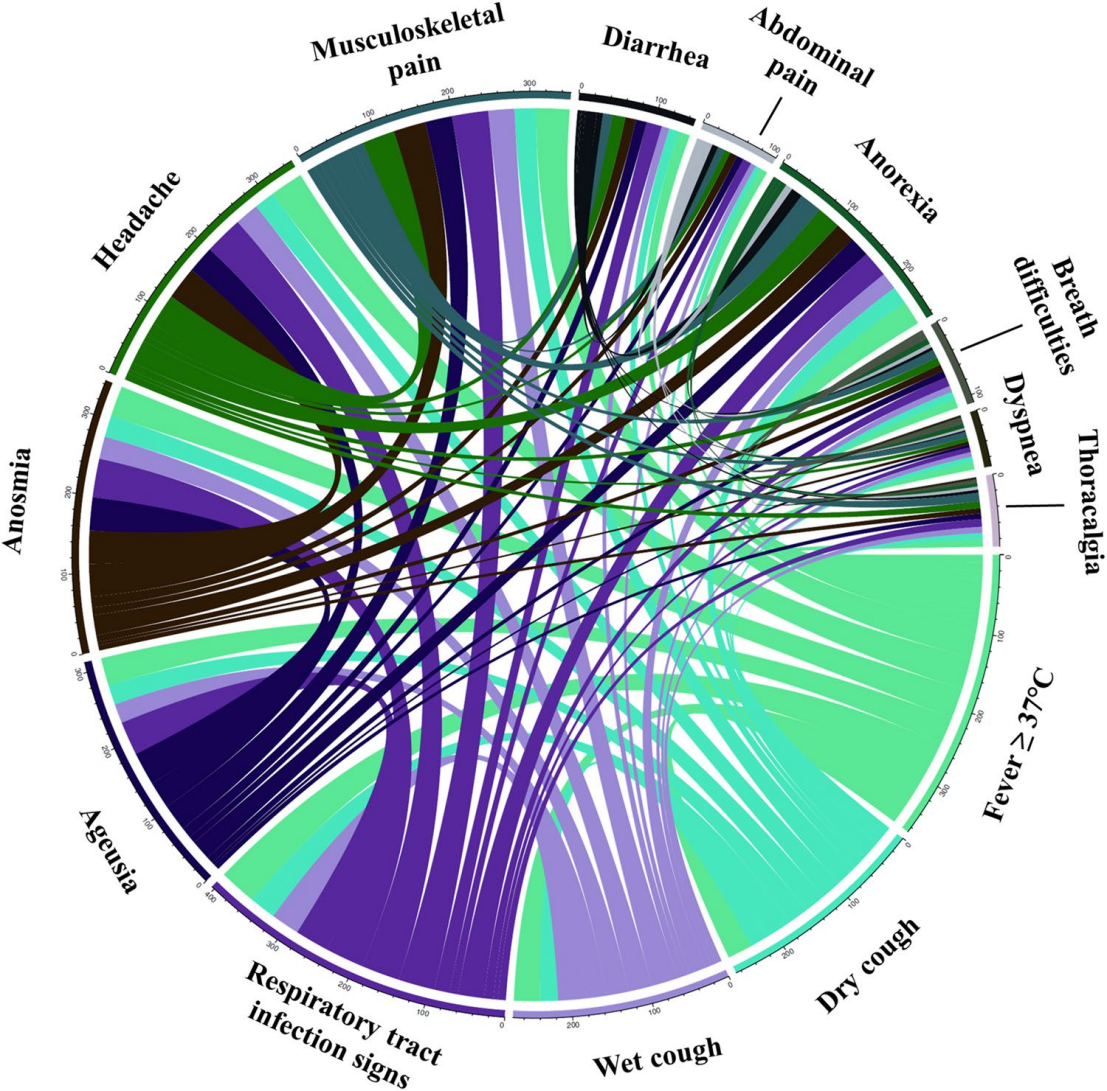
The frequency and co-occurrence of COVID-19 symptoms in outpatients. Circular visualization showing the frequency of symptoms’ co-occurrence. Arc length corresponds to the frequency of symptoms, whereas the width of the ribbons between 2 symptoms shows the frequency of co-occurrence. Only symptoms co-occurring in more than three patients were included.

**Figure 4:**
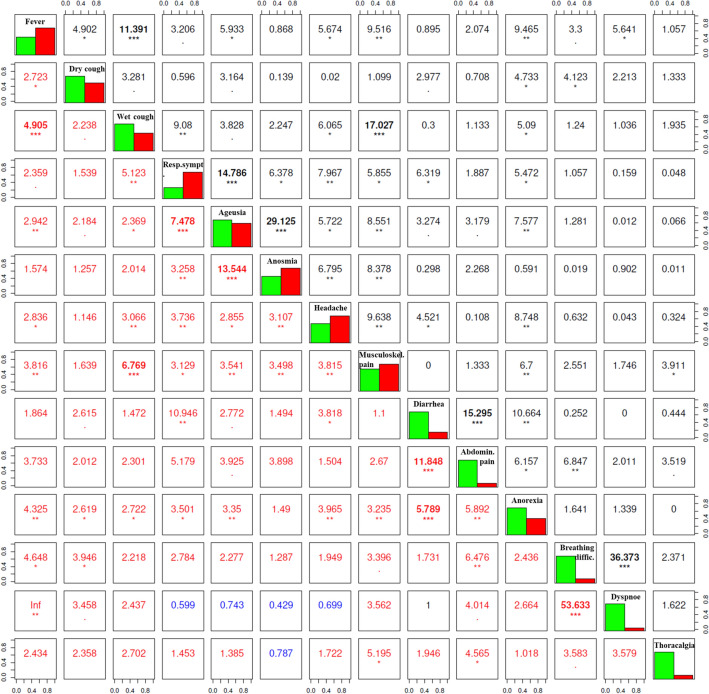
The correlation between COVID-19-symptoms’ co-occurrence in outpatients evaluated by using the Pearson’s Chi-squared tests (upper right) and Fisher’s odds ratio exact tests (lower left). Figure represents the relation between symptoms’ co-occurrence. The diagonal from the upper left corner to the lower right corner contains frequency histograms of each variable (green—symptom absent; red—symptom present). The Pearson’s Chi-squared tests (on the right top of the diagonal) measure the strength of a linear association between categorical variables presented by the Pearson correlation coefficient. The Fisher’s odds ratio exact tests (on the bottom left of the diagonal; red and blue numbers indicate positive and negative associations, respectively) represent the ordinal dependence between two measured quantities. Each significance level is depicted by stars: *p < 0.05; **p < 0.01; ***p < 0.001. Only symptoms co-occurring in more than three patients were included. *COVID-19* Coronavirus Disease 2019.

We recognized a statistically significant (p < 0.001) link between anosmia and ageusia, fever and wet cough, musculoskeletal pain and wet cough, general RTI symptoms and ageusia, diarrhea and abdominal pain, and breathing difficulties with dyspnea. The co-occurrence of clinical signs is recorded in Figs. 3 and 4.

#### Association between symptoms and comorbidities

**Figure 5:**
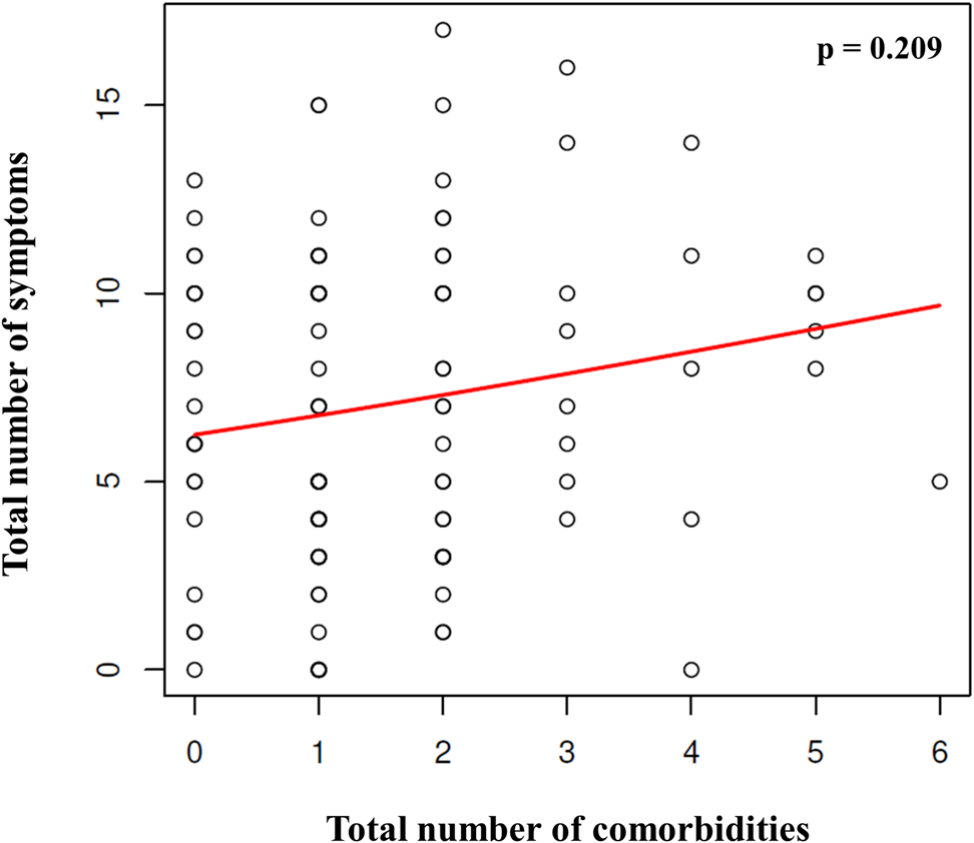
The relationship between comorbidities and the number of symptoms in outpatients. Figure represents the relationship between the total number of comorbidities and the total number of symptoms in outpatients (Spearman’s rank correlation r = 0.12, p = 0.21). One circle corresponds to a unique patient, however, the circles of some patients may overlap in the graph. The red line represents a non-parametric regression function.

We analyzed the relationship between patients' characteristics and comorbidities with the number of symptoms. In general, a linear relationship appeared between a higher number of comorbidities and a higher number of symptoms (p = 0.209) (Fig. 5). However, neither an older age (p = 0.077), female gender (p = 0.254), diabetes mellitus (p = 0.129), cancer (p = 0.699), nor allergies (p = 0.171) were determined statistically significantly correlated with the number of disease symptoms. Patients exhibiting fewer symptoms were those with a higher BMI (p = 0.370) and, surprisingly, smokers (p = 0.096), although this relationship was statistically insignificant. Hypertension did not affect the symptom number (p = 0.548), but anosmia incidence was higher in patients without arterial hypertension compared to patients having this comorbidity (40% vs. 65%; p = 0.036). There was no link between hypertension and ageusia presence. Neither anosmia nor ageusia were influenced by diabetes mellitus presence.

#### Sensory disorders with extended follow-up

By October 31, 2020, we had completed a detailed reassessment of sensory disorder incidence over time in our two negative test cohort with a median follow-up of 181 days (Table 7). Total median for anosmia and ageusia length was 32 days and 21 days, with persisting symptoms in 26% and 5% of outpatients, respectively. At the time of second negative SARS-CoV-2 PCR testing, anosmia and ageusia were present in almost half (48%) and a quarter of the outpatients (21%), respectively.

**Table 7:** Sensory disorders with extended follow-up in the cohort with two negative tests.

Total evaluated patients, N (%)	40 (100)
**Ageusia**	
No of patients with ageusia, n (%)	19 (47.5)
No of patients with ageusia at the time of the 2nd negative test, n (%)	4 (21.1)
Duration of ageusia (days), median (mean, min–max)	21 (37; 2–159)^a^
Duration of ageusia after the 1st negative test (days), median (mean, min–max)	0 (12; 0–131)
Duration of ageusia after the 2nd negative test (days), median (mean, min–max)	0 (12; 0–128)
**Anosmia**	
No of patients with anosmia, n (%)	23 (57.5)
No of patients with anosmia at the time of the 2nd negative test, n (%)	11 (47.8)
Duration of anosmia (days), median (mean, min–max)	32 (67; 2–194)^b^
Duration of anosmia after the 1st negative test (days), median (mean, min–max)	0 (44; 0–171)
Duration of anosmia after the 2nd negative test (days), median (mean, min–max)	0 (43; 0–169)

^a^1 patient still had ageusia at the time of follow-up (data adjusted to the date of the last follow-up—October, 31th, 2020).

^b^6 patients still had anosmia at the time of follow-up (data adjusted to the date of last follow-up—October, 31th, 2020).

#### Incidence of COVID-19 symptoms during extended follow-up

Although our analysis primarily focused on the initial phase of evaluating acute symptoms, we assessed the incidence of COVID-19 symptoms in outpatients during an extended monitoring study phase with a median follow-up of 219 days, which is detailed in Table 8.

**Table 8:** Incidence of COVID-19 symptoms among 66 outpatients during follow-up phase.

All outpatients (N = 66)				
Type of symptom	N (%)			
	M1	M2	M3	M6
Fatigue	8 (12)	2 (3)	1 (2)	1 (2)^a^
Fever ≥ 37 °C	4 (6)	0 (0)	0 (0)	0 (0)
Dry cough	8 (12)	6 (9)	5 (8)	3 (5)^a^
Wet cough	6 (9)	2 (3)	2 (3)	2 (3)^a^
Respiratory tract infection signs	5 (8)	2 (3)	1 (2)	0 (0)
Ageusia	11 (17)	5 (8)	4 (6)	3 (5)^a^
Anosmia	19 (29)	14 (21)	11 (17)	11 (17)^a^
Headache	3 (5)	2 (3)	1 (2)	1 (2)^a^
Musculoskeletal pain	3 (5)	0 (0)	0 (0)	0 (0)
Diarrhea	0 (0)	0 (0)	0 (0)	0 (0)
Abdominal pain	1 (2)	0 (0)	0 (0)	0 (0)
Anorexia	1 (2)	0 (0)	0 (0)	0 (0)
Vomiting	0 (0)	0 (0)	0 (0)	0 (0)
Breath difficulties	1 (2)	1 (2)	0 (0)	0 (0)
Dyspnea	1 (2)	1 (2)	0 (0)	0 (0)
Shortness of breath	0 (0)	0 (0)	0 (0)	0 (0)
Tachypnea	0 (0)	0 (0)	0 (0)	0 (0)
Thoracalgia	2 (3)	1 (2)	1 (2)	1 (2)^a^
Dry skin	1 (2)	1 (2)	1 (2)	0 (0)

Although our analysis primarily focused on acute symptomatology during the study’s initial phase, incidence of symptoms during an extended monitoring study phase with a median follow-up of 219 days (mean-222; min-32, max-486) is presented in Table 8. We evaluated in total 23 (58%) of the patients from our Cohort with 2 Negative Tests, and 43 (66%) of the patients from our Cohort with New Algorithm, respectively. During the first month following COVID-19 diagnosis, certain symptoms persisting with a frequency greater than 10% in outpatients were: anosmia (29%), ageusia (17%), fatigue (12%) and dry cough (12%), respectively. However, at the 6 month follow-up, only anosmia was detected with a higher frequency (17%). Assuming that patients no longer had a reason to deny symptoms after quarantine release, we conducted evaluations jointly for both cohorts. Only patients who agreed to enter the extended follow-up phase were included in our analysis.

*COVID-19* coronavirus disease-19, *M1, M2, M3, M6* number of months after COVID-19 diagnosis during follow-up phase.

^a^Certain patients continuing to evince symptoms at the time of follow-up.

### SARS-CoV-2 viral load

A total of 105 diagnostic (i.e. the first positive specimen in a unique patient) nasopharyngeal swabs were analyzed. Among the two negative tests cohort, a total of 148 follow up samples were examined (median 3; mean 3.7; min 2, max 9). Median diagnostic viral load was 25.1 Ct (mean 25.6; min 9.8, max 45.5) with viral load similar in both patient cohorts. In the group of 6 asymptomatic patients, median diagnostic viral load was 32.9 Ct (mean-32.4; min-18.9, max-41.7).

#### Correlation of viral load with symptoms during diagnosis

**Figure 6:**
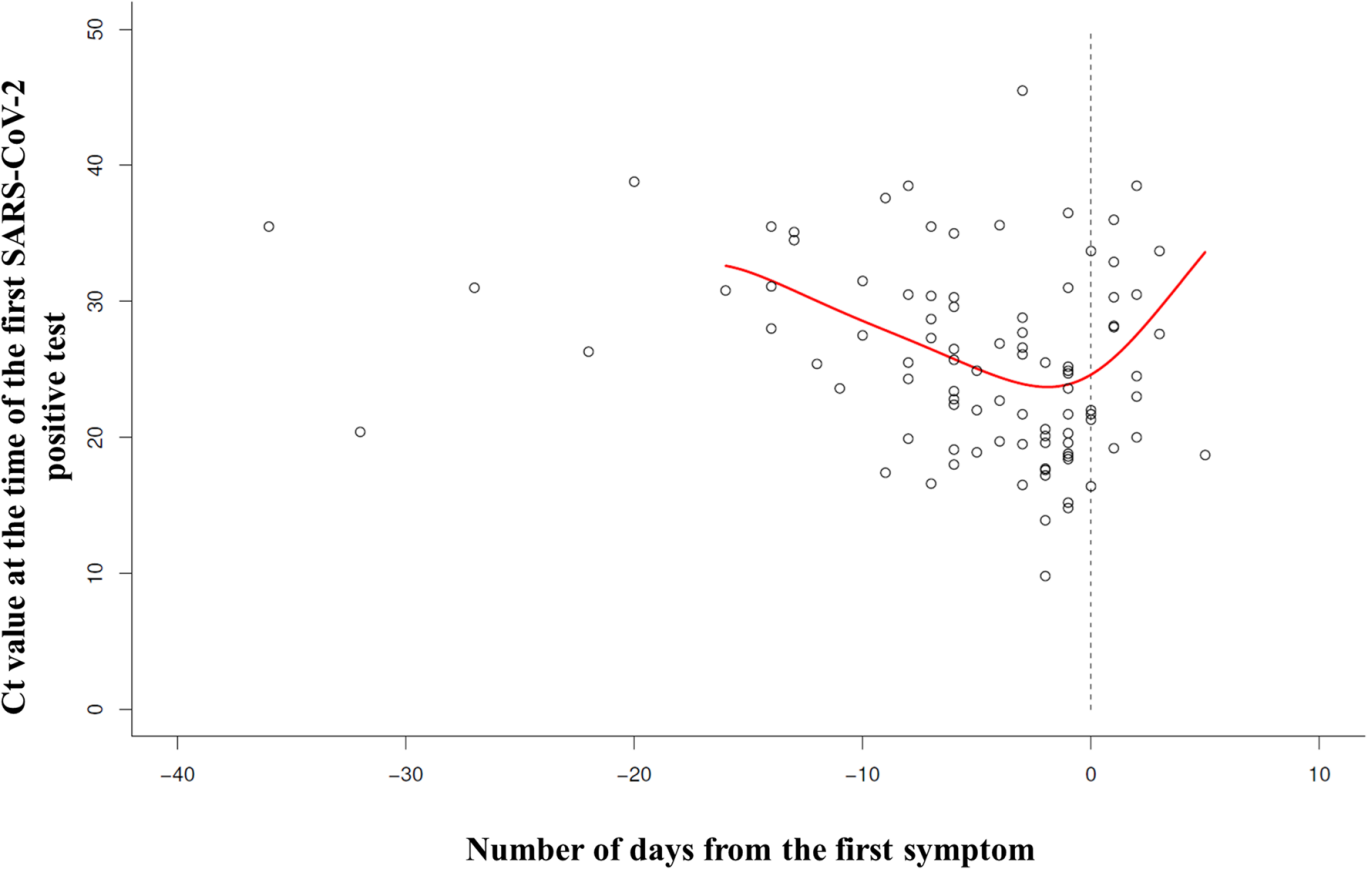
The correlation between Ct value of the first positive test and the time of initial symptom onset. Figure shows the correlation between diagnostic Ct value (performed on day 0) and the day of initial symptoms’ onset. Each small circle represents one unique symptomatic patient (the six completely asymptomatic patients were excluded from the analysis; additionally, another four patients with unknown absolute positive Ct value were excluded). To the right of the dashed line, a total of fourteen patients detected as contacts are shown (e.g. symptoms appeared after sampling). Patients already symptomatic at the time of the first positive PCR test sampling (N = 81) are displayed directly on the dashed line and to the left side of the dashed line. The correlation red curve between diagnostic Ct values and sampling time in relation to the symptoms’ onset is plotted U-shape. *Ct* cycle threshold, *PCR* polymerase chain reaction, *SARS-CoV-2* severe acute respiratory syndrome coronavirus 2.

We evaluated the correlation between diagnostic Ct value in relation to the time between sampling and first symptom onset (six patients with completely asymptomatic disease course were excluded from the analysis, while another four patients with unknown absolute positive Ct value were marginalized), see Fig. 6. Fourteen patients were affirmed as contacts up to 5 days before symptoms’ onset (i.e. symptoms appeared after sampling—the graph’s right portion). The remaining 81 patients, with known Ct value, were symptomatic at the time of their first positive PCR test, and they had already been symptomatic for an average of 6 days (i.e. symptoms appearing before sampling, left side of the graph). The maximum sampling time was 36 days after symptoms’ onset. Correlation curve plotted U-shape between diagnostic Ct values and sampling time in relation to the symptoms’ onset. Highest viral load was detected in diagnostic samples analyzed 0–2 days after initial symptom onset. Albeit not precisely recorded in numbers, patients rationalized during telemonitoring that delays between the symptom onset and sampling resulted from either being scared of COVID-19 positive diagnosis or by an insufficient testing capacity.

#### SARS-CoV-2 elimination course

**Figure 7:**
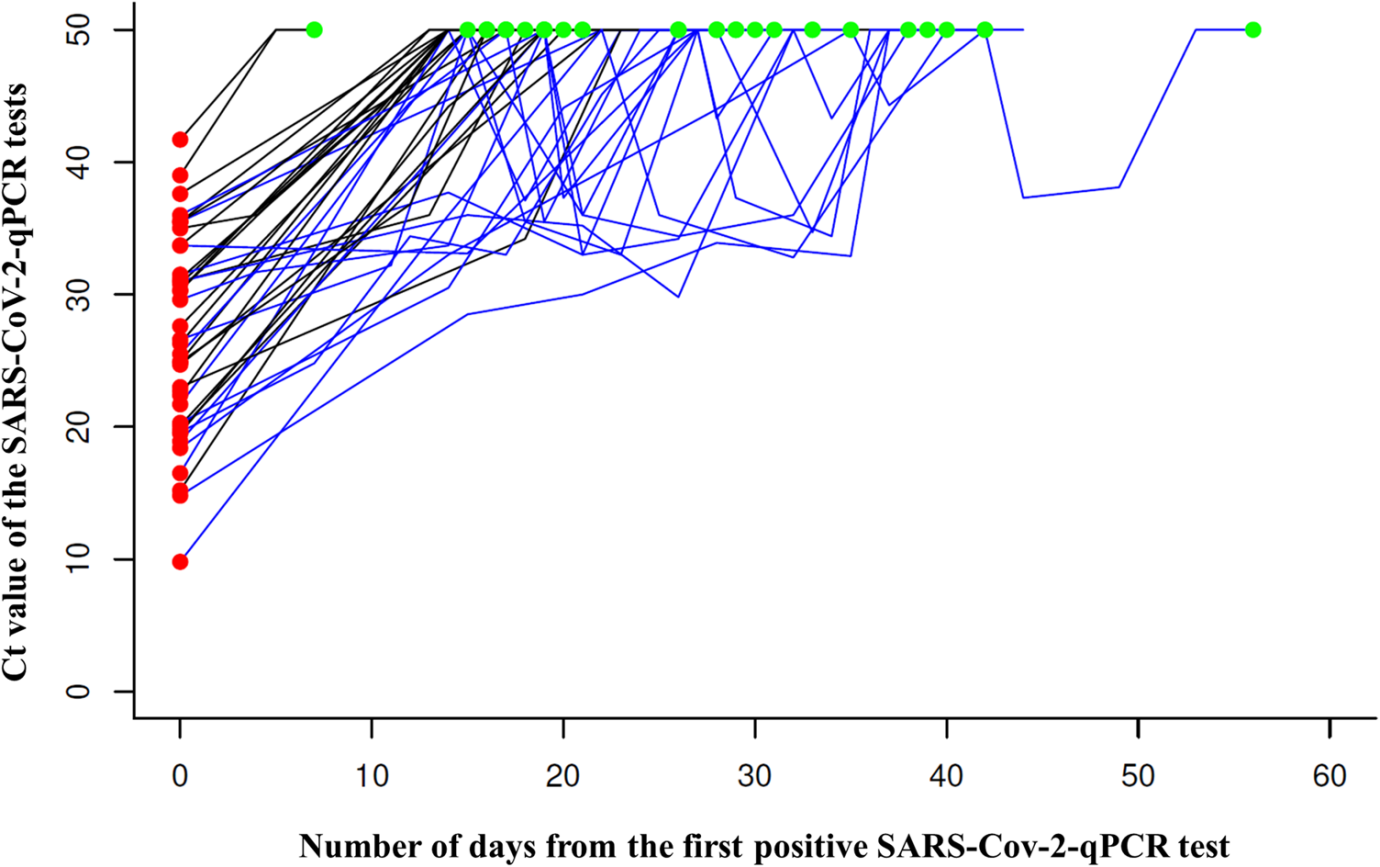
Viral load dynamics in the cohort with two negative tests. Figure illustrates the viral load dynamics of a total of 40 diagnostic (red points on the left side of the graph) and 148 follow-up samples evaluated in 40 patients from the cohort with two negative tests. The second negative tests are highlighted as green points at the top of the graph. Constantly increasing virus elimination curves are colored black, while fluctuating ones are blue. *SARS-CoV-2* severe acute respiratory syndrome coronavirus 2, *Ct* cycle threshold, *qPCR* quantitative polymerase chain reaction.

Among 40 patients from the two negative test cohort, median time from diagnostic sample to the first and the second negativity was 19 days (mean 21.9; min 5, max 53), and 26 days (mean 25.3; min 7, max 56), respectively. Median time from first to second negative sample was 2 days (mean 3.4; min 1, max 18). The virus elimination curve was steadily increasing (57%) or fluctuating (43%), see Fig. 7.

With this patient cohort, a total of 44% and 31% still exhibited symptoms at the time of the first and the second negative test, respectively. Yet, in the new algorithm cohort, only 21% of patients reported any symptom 11 days after the first positive test.

## Discussion

Despite organizing our study early in 2020, when there was far less knowledge about COVID-19, we feel our findings, nonetheless, are still quite relevant and important. Recruitment was intended to last for a few weeks. However, when disease incidence dramatically decreased following the “first wave”, cohort recruitment significantly subsided, allowing us an opportunity to specifically monitor and evaluate various symptoms during a longer follow-up period.

Within the Czech population, there was no significant difference in the frequency of symptoms compared to published data^3,4,12–15^. Regarding olfactory disorders, a pooled frequency differed between detection via smell testing (76%) and survey/questionnaire report (53%), which corresponds to our data (59%)^13^. In concordance with literature, we indicated an anosmia incidence higher in women and in younger patients^7^. However, our detailed monitoring subsequently revealed a spectrum of less frequent symptoms actually related to COVID-19 which should not be underestimated in practice (Table 6). These symptoms should now be included within the already published set of symptoms^3^.

In our study, precise mapping of symptom length represents a unique design, resulting from back tracing before the first PCR sampling. Notwithstanding our effort, we were unable to determine exact symptom duration in many patients owing to monitoring termination for double PCR negativity in the first cohort. Moreover, the second cohort of patients with the new algorithm of quarantine cessation probably shortened the symptoms deliberately. Despite no statistically significant differences in demographic parameters and comorbidities between these two cohorts, patients in our second cohort reported fewer symptoms, and much earlier, than the patients in the first cohort. For example, the differences in anosmia duration were striking. We may speculate that the motivation was to dissimulate non-severe symptoms in order to be released from quarantine as early as possible, since there was no requirement for PCR negativity at the end of quarantine. However, these unfit patients still, in fact, could represent a further source of virus spreading. To the best of our knowledge, such data detailing patient behavioral modification responding to COVID-19 quarantine regulations has not yet been published. On the other hand, the relative success of governmental measures depends heavily on a population’s willingness to actively participate. One report investigated perceived usefulness, adherence, and predictors of behavioral measures in eight countries and recognized significant differences. Some people felt particularly isolated and not well supported when certain regional governments postured ambivalent attitudes toward the measures, while in other countries, people deemed governmental communication quite positive^30^. With symptom duration definitely prolonged well beyond our telemonitoring capacity, further study of COVID-19 became justified. Nonetheless, specific symptoms definitely persisted even during double PCR negativity and/or monitoring termination, which we precisely documented. In our first cohort, a significant total of 44% and 31% of patients still exhibited some symptoms at the time of both first and second negative test, respectively. Along with anosmia and/or ageusia, symptoms included dry or wet cough, general RTI symptoms, headache, breathing difficulties, dyspnea, shortness of breath, thoracalgia, dry skin, and additional complications that developed during the disease. Furthermore, certain symptoms, particularly anosmia and ageusia, persisted during our extended phase for more than half a year after COVID-19 diagnosis. In literature, this topic is one of the most discussed issues regarding COVID-19. Persisting sensory dysfunction was observed in up to a quarter of of patients^31^. The mechanism of COVID-19 related olfactory dysfunction differed from those observed with an acute cold and may reflect a specific central nervous system impairment in some COVID-19 patients^32^. We believe, therefore, that government and health authority quarantine cessation guidelines need to reflect our factual findings. Current Czech Republic regulations mandate an early quarantine termination after just 10 days and without a negative test, when the patient is asymptomatic for the last 3 days^33^. Yet, specific mandatory guidelines regarding asymptomatic patient detection do not, however, presently exist. Moreover, recent emphasis seems to focus on discussing long-term consequences affecting particular patients^4,6,12,34^.

Based on contemporary data, no COVID-19 specific symptoms beyond smell and taste loss have been recorded^6,13^. In our study, 59%, 47%, and 65% of patients reported anosmia, ageusia, or both, respectively, while symptoms appeared on average 4.5 and 4.6 days, respectively, following disease onset. Thus, nearly two-thirds of patients are clinically detectable, albeit at the expense of regrettable delay, during which time the virus could spread unabated following disease onset.

Considering the co-occurrence of symptoms, we have confirmed a significant link between anosmia and ageusia^15^. Unlike large published data sets^35^, we have not, with our 105 patient cohort, established statistical significance in the correlation between particular symptoms and comorbidities, which emphasizes, in fact, that a given patient’s disease course is not dependably predictable in advance, which emphasizes the advantage of recommending an individualized telemedicine approach. This similarly holds true for predicting complications and the need for hospitalization. While certain risk factors have been detailed^25,36,37^, we concur that focused routine telemonitoring is a preferable option. Furthermore, our research has substantiated that COVID-19 symptoms can overlap with another disease, which emphasizes advantages of careful monitoring. The telemonitoring questionnaire we have designed is timely, applicable, and can be employed by paramedics as well as experienced professionals.

Severe documented pneumonia did not occur in our study with a high frequency (2%), which is reassuring. On the other hand, we quite often observed certain respiratory symptoms, which were not easily explainable (dyspnea, 10%; breathing difficulties, 13%; shortness of breath, 3%; tachypnea, 3%). Patients were not examined by auscultation, and monitoring staff did not consider symptoms severe enough to require a CT scan. Theoretically, we could have missed clinically mild COVID-19 pneumonias. In literature, pneumonia with a CT pathological determination was described in up to 100% of asymptomatic and pre-symptomatic patients^5,19–22^. Moreover, these patients exhibited longer virus shedding which might facilitate disease transmission^21^. Hence, we advocate the need for a well-designed study concerning chest CT examination in all newly diagnosed COVID-19 patients, which is highly important with respect to ethical and irradiation issues. Clearly corresponding with data for the Czech Republic (5.9%) during the observed period, only a small representative proportion of outpatients (5.7%) in our study required secondary hospitalization during their course of COVID-19.

Only a few people in our study (13%) were detected as contacts on average 2 days before the onset of symptoms, with only 6% of other patients being completely asymptomatic thru the course of the disease. Conversely, most patients (81%) were already symptomatic at the time of sampling, performed on day 0 up to day 36—on average day 6—which is relatively late in terms of disease onset. With the highest viral load at the time of initial symptom onset, as affirmed in our analysis (see Fig. 6), these patients exemplified massive SARS-CoV-2 spreaders. Our data places maximum emphasis on hygiene measures, wearing face masks, educating masses regarding symptoms, transmission and spread, and the imperative for early testing as quickly as possible^38^. According to interviews, patient delays resulted from fear of testing, fear of a COVID diagnosis, and/or insufficient testing capacity.

Recently, quite controversial yet very important topics have emerged regarding the relationship between PCR positivity duration, viral shedding, and the potential for infectivity, and possible reinfection, which is crucial for preventing virus spread and effective vaccine development^39–41^. A prospective extensive French study analyzing 3790 SARS-CoV-2 qPCR-positive nasopharyngeal samples and 1941 cell culture isolates has enriched knowledge about duration and frequency of live virus shedding^42^. Samples with Ct = 25 up to 70% positivity in virus culture were recognized. However, for samples with Ct 30, this ratio decreased to 20%, and at Ct 35, less than 3% of cultures were positive.

In addition, another prospective analysis evaluated potential infectivity not only in the correlation between Ct values and virus growth capacity in the cell culture but also by determining neutralizing antibodies in healthcare professionals with prolonged virus shedding up to 55 days^43^. Positive Ct values above 30 corresponded to non-viable particles. In the case of Ct-values below 30 and the simultaneous detection of neutralizing antibodies, authors also assumed non-infectivity. Literature review indicated that patients with severe-to-critical illness or those immunocompromised could shed the infectious virus for a significantly longer period than 1 month^44–46^.

The minimal viral load to be infected is unknown in humans and will probably vary among different people owing to many inherited and acquired factors. Moreover, culturable or non-culturable sample may not necessarily equal the real infectious capacity^45,46^. Nevertheless, as we determined, Ct values can vary during follow-up, which is an intriguing, previously reported phenomenon^45,46^ that has not yet been exactly explained. Usually, the presence of viral RNA without sample cultivability is interpreted as a non-vital virus shedding^46^. Keeping in mind potential serious social, emotional, and economic consequences of a longer quarantine along with rationale noted above, we would be very cautious regarding a fixed time interval quarantine for all patients. Moreover, we agree with Fontana et al.^46^, that further data is needed to understand the correlation between transmission risk, culture positivity, and Ct thresholds. In the Czech Republic, a second wave of the epidemy began upon the easing of very strict initial measures, which included quarantine up until a double negative PCR test.

## Conclusions

Our timely study has detailed COVID-19’s natural course among outpatients in terms of PCR-measured viral load kinetics. Disease course apparently is significantly variable, although with certain individuals, on the basis of comorbidities and other characteristics, trajectory may not be reliably predicted, emphasizing necessity for individualized patient monitoring and management. Double PCR negativity will not necessarily ensure simultaneous symptom disappearance.

Government regulations including prospective short fixed quarantine without a need for definitive PCR negativity might modulate patient behavior, influence individual reporting of non-serious symptoms, and eventually lead to inappropriate premature patient release from quarantine, thus contributing to further infection spread.

Only a minority of patients were expeditiously identified as COVID-19 contacts. Others, although symptomatic, were often detected following significant delays, which contributed to the virus spread. Individual viral kinetics displayed immense variability and fluctuated in nearly half of the patients.

Based on these findings, we recommend: (1) a wide-ranging intensive sophisticated precise, and long-term educational campaign focused on the entire population in order to diagnose the disease as early as possible, maximize tracing, and facilitate strict adherence to hygiene measures; (2) considering a more individualized model for quarantine termination; (3) improving communications between patients, general practitioners, and/or healthcare workers with follow-up telemonitoring in accordance with a predefined questionnaire aimed at early detection of potential complications and disparate serious diseases relating to acute COVID-19.

## Supplementary Information

Supplementary Information.
