# Supplementary material for: COVID-19’s natural course among ambulatory monitored outpatients
Source: Sci Rep. 2021 May 12;11:10124. doi: 10.1038/s41598-021-89545-1 (PMC8115337; doi:10.1038/s41598-021-89545-1)
Supplement: Supplementary file 1 — Supplementary Information. [file 41598_2021_89545_MOESM1_ESM.docx]

**COVID-19’s Natural Course Among Ambulatory Monitored Outpatients.**

Barbora Weinbergerova, Jiri Mayer, Stepan Hrabovsky, Zuzana Novakova, Zdenek Pospisil, Lucie Martykanova, Katerina Hortova, Lucie Mandelova, Karel Hejduk, Renata Chloupková, Michal Pospisil, Martina Doubkova, Vladimir Marek, Renata Novotna, Martin Dolecek, Hana Matejovska Kubesova, Kristian Brat, Radana Parizkova, Petr Husa, Marek Mechl, Zdenek Kral, and Martina Lengerova

**Predefined questionnaire**

This document characterizes an algorithm of a structured interview with a patient who has a COVID-19 positive lab result, is in isolation, has signed a consent to be included in a pilot project, signed a personal data agreement, and has thereby agreed to be enrolled into a COVID-JMK-20 study.

When a lab result is positive, information will be documented within a “Pilot Project Participation Consent” form which would then be submitted to the University Hospital Brno workforce in order to remotely contacted the patient.

On the first day, the person managing remote monitoring enters data (see below) recorded in the “Pilot Project Participation Consent” form into the local secured database.

**Entry data**

- Full name
- Personal identification number
- Address
- Phone number
- Attending physician ID
- SARS-CoV-2 diagnostic PCR test result including information about viral load quantification and lab ID

Additional, information regarding patient’s medical history will be included if accessible.

- Anamnesis
- Objective examination

Patient will be contacted daily for one week by phone via a number from the “Pilot Project Participation Consent” form. At the beginning of the initial contact, caller will briefly introduce the pilot project which patient agreed during testing to participate in and confirm that the patient is in isolation at home. Then, through incremental questioning, patient medical state will be monitored. After a brief review of the patient’s health condition, caller will focus on respiratory issues/complications.

**Actual clinical condition data:**

- Symptomatic/asymptomatic disease
- Body temperature (normal/fever ≥37°C)
- Cough (none/dry/wet)
- Respiratory tract infection (RTI) signs
- Sensory impairment (smell and taste)
- Headache
- Musculoskeletal pain
- Diarrhea
- Abdominal pain
- Anorexia
- Vomiting
- Dry skin
- Other symptoms

**Questions concerning breathing difficulties during every phone call:**

- Evaluating even minimal shortness of breath
- Dyspnea (subjective feeling of breathing difficulty)
- Thoracalgia (chest pain or chest pressure)
- Shortness of breath
- Tachypnea (feeling of air insufficiency, short inspiration with inadequate oxygen intake – normal breath frequency approximately 15 breath cycles per minute, expiration being not three times longer than inspiration)

Person calling records all data into a .xls document. In cases of breathing difficulties or other potentially severe patient complaints reported during phone calls, patient contact details would be provided to a physician from the University Hospital study team. Physician would contact patient, evaluate medical condition and consider further examination (e.g. lung CT scan, ECG) or hospitalization.

Telemonitoring phone calls with a patient would be undertaken daily during the first week. In the second week, phone calls would be made every other day while clinical conditions improve up until:

- A minimum 14-day quarantine including 2 consequent negative SARS-CoV-2 PCR tests with no symptoms for at least 3 consecutive days (during the initial period from 20 April 2020 to 7 July 2020)
- A minimum 14-day quarantine including at least 4 asymptomatic days without the need for PCR test negativity (during the period from 8 July 2020 to 2 September 2020)
- Hospitalization
- Patient waives cooperation
- Patient deceases

From the third week, telemonitoring calling frequency would be adjusted to twice weekly if patient’s clinical condition is improving.

**Fig. 1. Telemonitoring algorithm.**

**Initial patient data:**

- Basic demographic data
- Medical history
- Clinical examination record
- Attending physician ID
- SARS-CoV-2 diagnostic lab results including quantification

**Phone call**

**Week 1**

**Phone call every day:**

- Day 1:
  - Initial patient data
  - Actual clinical condition including breathing difficulties
- Day 2-7
  - Actual clinical condition including breathing difficulties

**Actual clinical condition data:**

- Symptomatic/asymptomatic disease
- Body temperature (normal/fever ≥37°C)
- Cough (none/dry/wet)
- Respiratory tract infection (RTI) signs
- Sensory impairment (smell and taste)
- Headache
- Musculoskeletal pain
- Diarrhea
- Abdominal pain
- Anorexia
- Vomiting
- Dry skin
- Other symptoms

**Breathing difficulties:**

- Asking about even minimal shortness of breath
- Dyspnea
- Thoracalgia
- Shortness of breath
- Tachypnea

*Clinical condition of the patient is the same or worse*

**Phone call every day:**

- - Actual clinical condition including breathing difficulties

*Clinical condition of the patient improves*

**Phone call every other day:**

- - Actual clinical condition including breathing difficulties

**Phone call**

**Week 2**

**Phone call**

**Week 3 and further**

**Phone call interval based on patient’s actual clinical condition:**

- - Actual clinical condition including breathing difficulties

**Any breathing difficulties present**

**Contacting a physician of the University Hospital study team phphysician**
